# Does *reductio ad absurdum* have a place in evidence-based medicine?

**DOI:** 10.1186/1741-7015-12-106

**Published:** 2014-06-25

**Authors:** Florian Naudet, Bruno Falissard

**Affiliations:** 1 INSERM, U669 Maison de Solenn, 97 Boulevard de Port Royal, Paris cedex 14, 75679, France; 2 Centre d’Investigation Clinique CIC-P INSERM 0203, Hôpital de Pontchaillou, Centre Hospitalier Universitaire de Rennes et Université de Rennes 1, Rennes, France; 3 Centre Hospitalier Guillaume Régnier, Service Hospitalo-Universitaire de Psychiatrie, Rennes, France; 4 Université Paris-Sud and Université Paris Descartes, UMR-S0669, Paris, France; 5 AP-HP, Hôpital Paul Brousse, Département de Santé Publique, Villejuif, France

**Keywords:** Epistemology, Evidence-based medicine, Publication bias, Reductio ad absurdum, Statistics

## Abstract

In a meta-analysis published in *BMC Medicine*, we explored whether evidence-based medicine can actually be sure that ‘sucrose = sucrose’ in the treatment of depression. This paper, based upon a *reductio ad absurdum*, addressed an epistemological question using a ‘scientific’ approach, and could be disconcerting as suggested by Cipriani and Geddes’ commentary. However, most papers are based upon a mixture of observations and discussions about sense and meaning. Ultimately, there is nothing more than a story, told with words or numbers. Randomised controlled trials provide information about average patients that do not exist. These results ignores an entire segment of therapeutics that plays a crucial role, namely care. This information is usually set out using a ‘grammar’ that is ambiguous, since statistical tests of hypothesis have raised epistemological questions that are not as yet solved. Moreover, many of these stories remain untold, and unpublished. For these reasons evidence-based medicine is a vehicle for many paradoxes and controversies. *Reductio ad absurdum* can be useful in precisely this case, to underline how and why the medical literature can sometimes give an impression of absurdity of this sort. Even if the data analysis in our paper was rather rhetorical, we agree that it should comply with the classic standards of reporting and we provide the important extra data that Cipriani and Geddes have requested.

Please see related articles: http://www.biomedcentral.com/1741-7015/11/230 and http://www.biomedcentral.com/1741-7015/12/105.

## Background

In our recent paper published in *BMC Medicine*[1], we explored in a thought-provoking manner whether science can actually be sure that ‘sucrose = sucrose’ in the treatment of major depressive disorder. This paper was an original piece of research based upon a *reductio ad absurdum*, but basically an essay about science and care with certain epistemological dimensions. We submitted it to stimulate reflection about the validity of scientific knowledge in medicine and we are glad that it has worked, with a very interesting commentary by Cipriani and Geddes on our published article [2].

Beyond addressing a complex and controversial issue within the field of antidepressant research, we believed that the form of this unusual paper raised an important issue implicitly suggested by Cipriani and Geddes’ comments: does *reductio ad absurdum* have a place in evidence-based medicine?

### Evidence-based stories

Cipriani and Geddes are right to point out that while our paper addressed an epistemological question we approached it ‘scientifically’ by conducting a systematic review and meta-analysis. Indeed, this can be considered as a challenge to the comfortable dualism at the basis of modern science: words are seen as being for philosophers and numbers for ‘hard’ scientists. We clearly militate for abandoning such a position, which sterilises the debate, especially in the field of psychiatric research [3]. To sum up our position on this point, we consider that in most papers, whether they come from social and human sciences or from ‘harder’ sciences, there is a mixture of observation (the core activity of science, present in the methods and results section of papers) and discussion about sense and meaning (more related to philosophy, and in general present in the discussion section). Ultimately, there is nothing more than a story, told with words or numbers, even if it can sometimes be an evidence-based story.

### Evidence-based stories concerning an imaginary average patient

To deal with the question of variability and randomness, randomised controlled trials (RCT) tell stories about average patients who, unfortunately or hopefully, do not exist in practice. Statistical inferences underpinning RCT conclusions concern expected values of random variables. In more human terms, these inferences involve the comparison of two or three run-of-the-mill patients (average), with blurred profiles (standard deviation). The story told by an RCT focuses on efficacy, sometimes effectiveness, and specifically on the pre-post difference in a very limited aspect of the average patient. This story does not tell much about the individual patient’s story that a clinician is faced with [4] and ignores an entire segment of therapeutics that plays a crucial role: care that draws on what we might call the patient’s ‘irrationality’, which has no place in mainstream evidence-based stories.

### Stories with ambiguous grammar

Natural languages are mainly bottom-up constructions and this means that stories have more or less the same meaning for everybody that reads them. This is not true for evidenced-based stories, which rely on a top-down grammar that is substantially misunderstood. Indeed, statistical tests of hypotheses have raised epistemological questions that are not yet solved [5]. Statistical tests can be used for ‘behavioural inference’ (the perspective proposed by Neyman and Pearson) or for ‘inductive inference’ (the Fisher perspective). In the first situation, type-one error is considered, in the second, the *P*-value. Unfortunately, most clinicians and non-statistician researchers are in the habit of mixing the two approaches, and this creates fuzziness in most conclusions in biomedical research in general, and RCTs in particular. The importance of significance testing in science is surely overstated, with a fallacious tendency to deflect attention from the actual size of an effect [6,7] and dramatic suggestive power. Translated into clinical practice, statistical significance converts into an argument of authority.

### Family secrets and storytellers

Most families have secrets, but the kind and importance vary. When evidence-based stories are told, the storytellers at the top of the paper are not always a good indication of who actually wrote them, since ghost-writing is endemic [8]. Moreover, many evidence-based stories remain untold, especially when they do not convey a positive picture [9]. The highly competitive environment for researchers’ funding and career promotion, authors’ own ideological conflicts of interest, and the tendency by editors, reviewers and readers to be ‘scientific novelty-seekers’, select the newest and most attractive storylines [10]. The recent contestation of the European Medicine Agency’s plans for sharing data from clinical trials by AbbVie and InterMune [11] has illustrated how untold stories can be explicitly considered as trade secrets.

‘Family secrets’ can sometimes cause illnesses and scientific remedies cannot be the cure in the case of Evidence Based Stories. Europe has recently made an important step forward by voting in favour of a law that will require all clinical drug trials in Europe to be registered and their results reported in a public database [12].

### Absurd stories and *reductio ad absurdum*

For these reasons, and despite a grammar based upon probability, evidence-based medicine boasts more than its fair share of improbable stories. All the ingredients listed above are present in antidepressant literature, resulting in many paradoxes and controversies, based upon different ways of telling the same stories [13,14]. *Reductio ad absurdum* can be precisely useful in this case, to underline how and why medical literature can give an impression of absurdity of this sort. In evidence-based medicine, this manner of reasoning is usually confined to Christmas issues in general journals. This curious ritual probably has an outlet function, authorising a return of repressed material from evidence-based medicine in a socially acceptable manner.

## Conclusion

We are glad that the *BMC Medicine* editors and reviewers accepted our improbable story in which the data analysis was in fact rather rhetorical (as indeed are all evidence-based stories). We agree that it should nonetheless comply with the classic standards of story-telling [15] and we are pleased to provide the important extra data that Cipriani and Geddes asked for in two files (Additional files 1 and 2).

## Abbreviation

RCT: Randomised controlled trials.

## Competing interests

The authors declare that they have no competing interests.

## Authors’ information

NF is a psychiatrist who works as a chief resident at the Department of Psychiatry of the University of Rennes. His main research focuses on methodological peculiarities of the evaluation of treatments in psychiatry. FB is a psychiatrist and professor in biostatistics at the Université Paris-Sud. He works on subjective measurements and more generally in innovative methods that can be used to grasp the patient’s perspective.

## Supplementary Material

Additional file 1 DATABASE. Click here for file

Additional file 2 FORESTPLOT. Click here for file
