# Supplementary material for: Does reductio ad absurdum have a place in evidence-based medicine?
Source: BMC Med. 2014 Jun 25;12:106. doi: 10.1186/1741-7015-12-106 (PMC4070092; doi:10.1186/1741-7015-12-106)
Supplement: Additional file 1 — DATABASE. [file 1741-7015-12-106-S1.pdf]

RESPONSE	REMISSION	NAME	YEAR	TOTAL Placebo	TOTAL Fluoxetine	Total Venlafaxine	Responders placebo	Responders fluoxetine	Responders venlafaxine	Remiters placebo	Remiters fluoxetine	Remiters venlafaxine	ANXIOUS	DURATION	FUNDING BODY	FOLLOW-UP	Placebo responders	Type of patients	Scale	INIT PLACEBO	INIT FLUOXETINE	INIT VENLAFAXINE	Analysis	Mean age	Doses
YES	YES	WXL101497	2004	199		189	91		120	63		94	NO	8	GSK	7	Yes	Outpatient	MADRS	30,4		30	ITTLOCF	42	Variable
YES	YES	AK130940	2004	189		198	91		127	71		109	NO	8	GSK	7	Yes	Outpatient	MADRS	30,6		30,1	ITTLOCF	44	Variable
YES	NO	MY-1043/BRL	1991	118	289		60	167					NO	12	GSK	Missing	Yes	Outpatient	HAMD		Missing	Missing	ITTLOCF	42	Variable
YES	NO	Cunningham	1997	100		193	30		114				NO	12	Wyeth Ayerst	8	Yes	Outpatient	HAM-21	24,9		24,25	ITTLOCF	40	Variable
YES	NO	Cunningham	1994	76		72	42		52				NO	6	Wyeth Ayerst	6	Yes	Missing	HAM-21	24,41		25,02	ITTLOCF	41	Variable
NO	YES	Guelfi	1995	47		46	12			6		12	NO	4	Wyeth Ayerst	6	Yes	Inpatient	MADRS	34,9		35,5	ITTLOCF	56	Variable
YES	NO	Khan	1998	98		286	34		146				NO	12	Wyeth Ayerst	8	Yes	Outpatient	HAMD-21	24,71		24,71	ITTLOCF	42	Variable
YES	NO	Lecrubier	1997	76		78	48		65				NO	13	Wyeth Ayerst	8	Yes	Primary_care	MADRS	24,2		24,9	ITTLOCF	40	Variable
YES	NO	Mendels	1993	78		234	38		145				NO	6	Wyeth Ayerst	6	Yes	Outpatient	HAMD-21	Missing		Missing	ITTLOCF	39	Variable
YES	NO	Rudolph	1998	92		231	28		117				NO	6	Wyeth Ayerst	6	Yes	Outpatient	HAMD-21	25,6		25,5	ITTLOCF	43	Variable
YES	YES	Thase	1997	102		95	29		53	19		32	NO	8	Wyeth Ayerst	5	Yes	Outpatient	HAMD-21	24,1		24,1	ITTLOCF	41	Variable
YES	YES	Rudolph	1999	98	103	100	41	51	54	18	23	35	NO	8	Wyeth Ayerst	7	Yes	Outpatient	HAMD-21	25	26	25	ITTLOCF	40	Variable
YES	YES	Nemeroff	2007	102	104	102	37	45	51	22	28	31	NO	6	Wyeth Ayerst	6	Yes	Outpatient	HAMD-21	23,7	23,7	23,5	ITTLOCF	40	Variable
YES	YES	Sheehan	2009	95	99	95	35	35	47	16	17	24	NO	6	Wyeth Pharmaceuticals	8	Yes	Inpatient	HAMD-21	29,42	29,5	29,9	ITTLOCF	39	Variable
YES	YES	Silverstone	1999	118	119	122	50	74	80	28	54	56	YES	12	Wyeth Ayerst	8	Yes	Outpatient	HAMD-21	27,1	27	27,6	ITTLOCF	42	Variable
YES	YES	Alves	1999		47	40		35	35		5	12	NO	12	Wyeth Ayerst	9	Yes	Outpatient	HAMD-21		26,9	27,9	ITTLOCF	43	Variable
YES	NO	Clerc	1994		34	34		17	23				NO	6	Wyeth Ayerst	8	No	Inpatient	MADRS		35,7	34,8	ITTLOCF	52	200 mg V 40 mg F
YES	YES	Costa e Silva	1998		186	196		152	170		112	118	NO	8	Wyeth Ayerst	7	Yes	Outpatient	HAMD-19		29,7	30,4	ITTLOCF	40	Variable
YES	YES	De Nayer	2002		73	73		34	48		27	38	YES	12	Wyeth Lederle	Missing	No	Outpatient	HAMD-20		23,1	23	ITTLOCF	42	Variable
YES	NO	Dierick	1996		161	153		95	107				NO	8	Wyeth Ayerst	6	Yes	Outpatient	HAMD-21		26,8	26,8	ITTLOCF	43	Variable
YES	YES	Keller	2007		267	781		210	612		132	380	NO	10	Wyeth Research	8	Yes	Outpatient	HAMD-17		23	22,6	ITTLOCF	40	Variable
YES	YES	Tzanakaki	2000		54	55		36	39		19	22	NO	6	Wyeth Ayerst	6	Yes	Inpatient	MADRS		37	36,4	ITTLOCF	48	Variable
YES	YES	Tylee	1997		170	171		121	110		58	61	NO	12	Wyeth Laboratory	6	No	Primary_care	MADRS		29,2	27,8	ITTLOCF	44	75 mg V 20 mg F
YES	YES	Heiligenstein	1993	28	24		11	15		10	15		NO	8	Eli Lilly	9	Yes	Outpatient	HAMD-17	26,4	24,9		ITTLOCF	40	20 mg
YES	YES	Moreno	2005	26	20		11	11		9	9		NO	8	Eli Lilly / Marjan	9	Yes	Outpatient	HAMD-21	16,3	15,2		ITTLOCF	40	20 mg
YES	YES	Andreoli	2002	128	127		43	72		34	57		NO	8	Missing	9	No	Both	HAMD-21	27,4	26,9		ITTLOCF	42	Variable
YES	YES	Bjerkenstedt	2004	58	57		21	20		4	15		NO	4	Lichtwer pharma	3	Yes	Primary_care	HAMD-21	25,2	23,8		ITTLOCF	50	20 mg
YES	NO	Corrigan	2000	35	35		9	17					NO	8	Pharmacia and Upjohn	9	No	Missing	MADRS	26,8	28,8		ITTLOCF	42	20 mg
NO	YES	Fava	2005	43	47					9	14		NO	12	Lichtwer pharma	7	Yes	Outpatient	HAMD-17	19,9	19,6		ITTLOCF	37	20 mg
YES	NO	Fava	1998	19	54		10	31					YES	12	Smith Kline	8	Yes	Outpatient	HAMD-21	23,7	23,9		ITTLOCF	41	Variable
YES	YES	Goldstein	2002	70	33		33	17		22	10		NO	8	Eli Lilly	9	Missing	Outpatient	HAMD-17	19,2	17,9		ITTMM	40	20 mg

LEGEND	
**Response**	Studies are included in the response analysis
**Remission**	Studies are included in the remission analysis
**NAME**	Name of the first author or of the study if unpublished
**Year**	Publication year or year of the study if unpublished
**TOTAL Placebo**	Number of patient in the placebo arm
**TOTAL Fluoxetine**	Number of patient in the fluoxetine arm
**Total Venlafaxine**	Number of patient in the venlafaxine arm
**Responders placebo**	Number of responders in the placebo arm
**Responders fluoxetine**	Number of responders in the fluoxetine arm
**Responders venlafaxine**	Number of responders in the venlafaxine arm
**Remiters placebo**	Number of remiters in the placebo arm
**Remiters fluoxetine**	Number of remiters in the fluoxetine arm
**Remiters venlafaxine**	Number of remiters in the venlafaxine arm
**ANXIOUS**	Did the study concerned anxious depression
**DURATION**	Study duration
**Follow-up**	Number of follow-up assesments
**Placebo responders**	Was there an exclusion of placebo responders
**Type of patients**	Type of included patients
**Scale**	Type of scale used
**INIT PLACEBO**	Mean initial severity in the placebo arm
**INIT FLUOXETINE**	Mean initial severity in the fluoxetine arm
**INIT VENLAFAXINE**	Mean initial severity in the venlafaxine arm
**Analysis**	Type of analysis used (ITTLOCF : intention to treat with last observation carried forward / ITTMM : intention to treat with a mixed model)
**Age**	Mean age of the participants
**Doses**	Doses used in the study (V : venlafaxine, F : Fluoxetine, variable : different doses were possible in the study)

REFERENCES

WXL101497
AK130940
MY-1043/BRL-029060/115
Cunningham LA (1997) Once-daily venlafaxine extended release (XR) and venlafaxine immediate release (IR) in outpatients with major depression. Venlafaxine XR 208 Study Group. Ann Clin Psychiatry 9: 157-16
Cunningham LA, Borison RL, Carman JS, Chouinard G, Crowder JE, et al. (1994) A comparison of venlafaxine, trazodone, and placebo in major depression. J Clin Psychopharmacol 14: 99-10
Guelfi JD, White C, Hackett D, Guichoux JY, Magni G (1995) Effectiveness of venlafaxine in patients hospitalized for major depression and melancholia. J Clin Psychiatry 56: 450-45
Khan A, Upton GV, Rudolph RL, Entsuah R, Leventer SM (1998) The use of venlafaxine in the treatment of major depression and major depression associated with anxiety: a dose-response study. Venlafaxine Investigator Study Group. J Clin Psychopharmacol 18: 19-
Lecrubier Y, Bourin M, Moon CA, Schifano F, Blanchard C, et al. (1997) Efficacy of venlafaxine in depressive illness in general practice. Acta Psychiatr Scand 95: 485-49
Mendels J, Johnston R, Mattes J, Riesenberg R (1993) Efficacy and safety of b.i.d. doses of venlafaxine in a dose-response study. Psychopharmacology bulletin 29: 169-17
Rudolph RL, Fabre LF, Feighner JP, Rickels K, Entsuah R, et al. (1998) A randomized, placebo-controlled, dose-response trial of venlafaxine hydrochloride in the treatment of major depression. J Clin Psychiatry 59: 116-12
Thase ME (1997) Efficacy and tolerability of once-daily venlafaxine extended release (XR) in outpatients with major depression. The Venlafaxine XR 209 Study Group. J Clin Psychiatry 58: 393-39
Rudolph RL, Feiger AD (1999) A double-blind, randomized, placebo-controlled trial of once-daily venlafaxine extended release (XR) and fluoxetine for the treatment of depression. J Affect Disord 56: 171-18
Nemeroff CB, Thase ME, Group ES (2007) A double-blind, placebo-controlled comparison of venlafaxine and fluoxetine treatment in depressed outpatients. Journal of psychiatric research 41: 351-35
Sheehan DV, Nemeroff CB, Thase ME, Entsuah R (2009) Placebo-controlled inpatient comparison of venlafaxine and fluoxetine for the treatment of major depression with melancholic features. pp. 61-8
Silverstone PH, Ravindran A (1999) Once-daily venlafaxine extended release (XR) compared with fluoxetine in outpatients with depression and anxiety. Journal of Clinical Psychiatry 60: 22-2
Alves C, Cachola I, Brandao J (1999) Efficacy and tolerability of venlafaxine and fluoxetine in outpatients with major depression. Primary Care Psychiatry 5: 57-6
Clerc GE, Ruimy P, Verdeau-Pailles J (1994) A double-blind comparison of venlafaxine and fluoxetine in patients hospitalized for major depression and melancholia. International Clinical Psychopharmacology 9: 139-14
Costa e Silva J (1998) Randomized, double-blind comparison of venlafaxine and fluoxetine in outpatients with major depression. The Journal of clinical psychiatry 59: 352-35
De Nayer A, Geerts S, Ruelens L, Schittecatte M, De Bleeker E, et al. (2002) Venlafaxine compared with fluoxetine in outpatients with depression and concomitant anxiety. Int J Neuropsychopharmacol 5: 115-1
Dierick M, Ravizza L, Realini R, Martin A (1996) A double-blind comparison of venlafaxine and fluoxetine for treatment of major depression in outpatients. Prog Neuropsychopharmacol Biol Psychiatry 20: 57-7
Keller MB et al. The Prevention of Recurrent Episodes of Depression with Venlafaxine for Two Years (PREVENT) study: outcomes from the acute and continuation phases. Biol Psychiatry. 2007 Dec 15;62(12):1371-9. Epub 2007 Sep 7. Erratum in: Biol Psychiatry. 2008 Apr;63(7):721. Biol Psychiatry. 2012 Feb 15;71(4):3
Tzanakaki M, Guazzelli M, Nimatoudis I, Zissis NP, Smeraldi E, et al. (2000) Increased remission rates with venlafaxine compared with fluoxetine in hospitalized patients with major depression and melancholia. Int Clin Psychopharmacol 15: 29-
Tylee A, Beaumont G, Bowden MW, Reynolds A (1997) A double-blind, randomized, 12-week comparison study of the safety and efficacy of venlafaxine and fluoxetine in moderate to severe major depression in general practice. Primary Care Psychiatry 3: 51-
Heiligenstein JH, Tollefson GD, Faries DE (1993) A double-blind trial of fluoxetine, 20 mg, and placebo in out-patients with DSM-III-R major depression and melancholia. International clinical psychopharmacology 8: 247-2
Moreno RA, Teng CT, Almeida KM, Tavares Junior H (2005) Hypericum perforatum versus fluoxetine in the treatment of mild to moderate depression: a randomized double-blind trial in a Brazilian sample. Revista brasileira de psiquiatria 28: 29-
Andreoli V, Caillard V, Deo RS, Rybakowski JK, Versiani M (2002) Reboxetine, a new noradrenaline selective antidepressant, is at least as effective as fluoxetine in the treatment of depression. J Clin Psychopharmacol 22: 393-3
Bjerkenstedt L, Edman GV, Alken RG, Mannel M (2005) Hypericum extract LI 160 and fluoxetine in mild to moderate depression: a randomized, placebo-controlled multi-center study in outpatients. Eur Arch Psychiatry Clin Neurosci 255: 40-
Corrigan MH, Denahan AQ, Wright CE, Ragual RJ, Evans DL (2000) Comparison of pramipexole, fluoxetine, and placebo in patients with major depression. Depress Anxiety 11: 58-6
Fava M, Alpert J, Nierenberg AA, Mischoulon D, Otto MW, et al. (2005) A Double-blind, randomized trial of St John's wort, fluoxetine, and placebo in major depressive disorder. J Clin Psychopharmacol 25: 441-44
Fava M, Amsterdam JD, Deltito JA, Salzman C, Schwaller M, et al. (1998) A double-blind study of paroxetine, fluoxetine, and placebo in outpatients with major depression. Ann Clin Psychiatry 10: 145-1
Goldstein DJ, Mallinckrodt C, Lu Y, Demitrack MA (2002) Duloxetine in the treatment of major depressive disorder: a double-blind clinical trial. J Clin Psychiatry 63: 225-2
