# Supplementary material for: Does reductio ad absurdum have a place in evidence-based medicine?
Source: BMC Med. 2014 Jun 25;12:106. doi: 10.1186/1741-7015-12-106 (PMC4070092; doi:10.1186/1741-7015-12-106)

Placebo VS Fluoxetine : Response

Study	Placebo Events	Placebo Total	Fluoxetine Events	Fluoxetine Total	OR	95%-CI	W(fixed)	W(random)
MY-1043/BRL-029060/115	60	118	167	289	0.76	[0.49; 1.16]	17%	15.8%
Rudolph	41	98	51	103	0.73	[0.42; 1.28]	10.4%	11.1%
Nemeroff	37	102	45	104	0.75	[0.43; 1.31]	10.2%	11%
Sheehan	35	95	35	99	1.07	[0.59; 1.92]	7.7%	10.3%
Silverstone	50	118	74	119	0.45	[0.27; 0.75]	15.2%	12.2%
Heiligenstein	11	28	15	24	0.39	[0.13; 1.19]	3.5%	3.4%
Moreno	11	26	11	20	0.60	[0.19; 1.94]	2.6%	3.1%
Andreoli	43	128	72	127	0.39	[0.23; 0.64]	17.2%	12.7%
Bjerkenstedt	21	58	20	57	1.05	[0.49; 2.25]	4.6%	6.7%
Corrigan	9	35	17	35	0.37	[0.13; 1.00]	4.5%	4.1%
Fava	10	19	31	54	0.82	[0.29; 2.36]	2.7%	3.8%
Goldstein	33	70	17	33	0.84	[0.37; 1.92]	4.4%	5.8%
**Fixed effect model**	**361**	**895**	**555**	**1064**	**0.65**	**[0.54; 0.78]**	**100%**	**--**
**Random effects model**					**0.65**	**[0.53; 0.81]**	**--**	**100%**

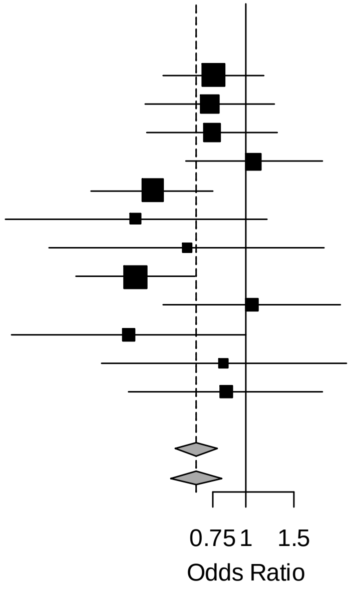

Placebo VS Venlafaxine : Response

Study	Placebo Events	Placebo Total	Venlafaxine Events	Venlafaxine Total	OR	95%-CI	W(fixed)	W(random)
WXL101497	91	199	120	189	0.48	[0.32; 0.73]	12.3%	12.7%
AK130940	91	189	127	198	0.52	[0.35; 0.78]	11.8%	12.7%
Cunningham	30	100	114	193	0.30	[0.18; 0.50]	10%	7.9%
Cunningham	42	76	52	72	0.48	[0.24; 0.94]	4.4%	4.5%
Khan	34	98	146	286	0.51	[0.32; 0.82]	9%	9.3%
Lecrubier	48	76	65	78	0.34	[0.16; 0.73]	4.4%	3.7%
Mendels	38	78	145	234	0.58	[0.35; 0.98]	6.8%	7.9%
Rudolph	28	92	117	231	0.43	[0.26; 0.71]	8.5%	8%
Thase	29	102	53	95	0.31	[0.17; 0.57]	7.2%	6%
Rudolph	41	98	54	100	0.61	[0.35; 1.07]	5.7%	6.7%
Nemeroff	37	102	51	102	0.57	[0.33; 1.00]	6%	6.7%
Sheehan	35	95	47	95	0.60	[0.33; 1.06]	5.5%	6.3%
Silverstone	50	118	80	122	0.39	[0.23; 0.65]	8.3%	7.7%
**Fixed effect model**	**594**	**1423**	**1171**	**1995**	**0.47**	**[0.40; 0.54]**	**100%**	**--**
**Random effects model**					**0.47**	**[0.40; 0.54]**	**--**	**100%**

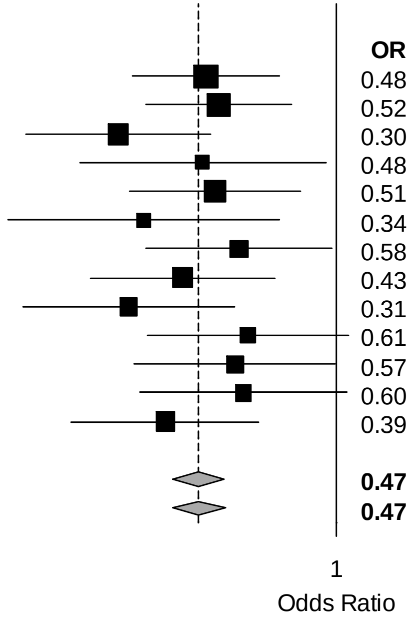

Fluoxetine VS Venlafaxine : Response

	Fluoxetine		Venlafaxine					
**Study**	**Events**	**Total**	**Events**	**Total**	**OR**	**95%-CI**	**W(fixed)**	**W(random)**
Rudolph	51	103	54	100	0.84	[0.48; 1.45]	7.9%	8.8%
Nemeroff	45	104	51	102	0.76	[0.44; 1.32]	8.3%	8.9%
Sheehan	35	99	47	95	0.56	[0.31; 0.99]	8.8%	8.3%
Silverstone	74	119	80	122	0.86	[0.51; 1.46]	8.5%	9.4%
Alves	35	47	35	40	0.42	[0.13; 1.31]	2.8%	2.6%
Clerc	17	34	23	34	0.48	[0.18; 1.28]	3.3%	3.5%
Costa e Silva	152	186	170	196	0.68	[0.39; 1.19]	8.6%	8.7%
De Nayer	34	73	48	73	0.45	[0.23; 0.88]	7.3%	6.6%
Dierick	95	161	107	153	0.62	[0.39; 0.99]	12.8%	11%
Keller	210	267	612	781	1.02	[0.73; 1.43]	19%	15.8%
Tzanakaki	36	54	39	55	0.82	[0.36; 1.85]	3.7%	4.8%
Tylee	121	170	110	171	1.37	[0.87; 2.16]	9%	11.4%
**Fixed effect model**	**905**	**1417**	**1376**	**1922**	**0.80**	**[0.68; 0.93]**	**100%**	**--**
**Random effects model**					**0.77**	**[0.63; 0.94]**	**--**	**100%**

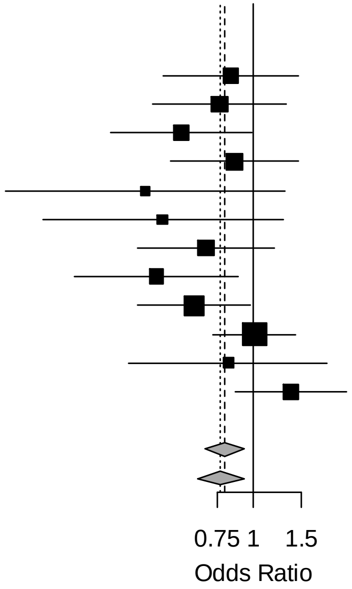

Placebo VS Fluoxetine : Remission

Study	Placebo Events	Placebo Total	Fluoxetine Events	Fluoxetine Total	OR	95%-CI	W(fixed)	W(random)
Rudolph	18	98	23	103	0.78	[0.39; 1.56]	9.7%	12%
Nemeroff	22	102	28	104	0.75	[0.39; 1.42]	11.6%	13.3%
Sheehan	16	95	17	99	0.98	[0.46; 2.07]	7.4%	10.7%
Silverstone	28	118	54	119	0.37	[0.21; 0.65]	21.8%	15.9%
Heiligenstein	10	28	15	24	0.33	[0.11; 1.03]	5.5%	5.5%
Moreno	9	26	9	20	0.65	[0.20; 2.14]	3.5%	5%
Andreoli	34	128	57	127	0.44	[0.26; 0.75]	22.4%	17.1%
Bjerkenstedt	4	58	15	57	0.21	[0.06; 0.67]	7.5%	5.2%
Fava	9	43	14	47	0.62	[0.24; 1.64]	5.6%	7.2%
Goldstein	22	70	10	33	1.05	[0.43; 2.59]	5%	8.1%
**Fixed effect model**	**172**	**766**	**242**	**733**	**0.56**	**[0.44; 0.71]**	**100%**	**--**
**Random effects model**					**0.57**	**[0.43; 0.76]**	**--**	**100%**

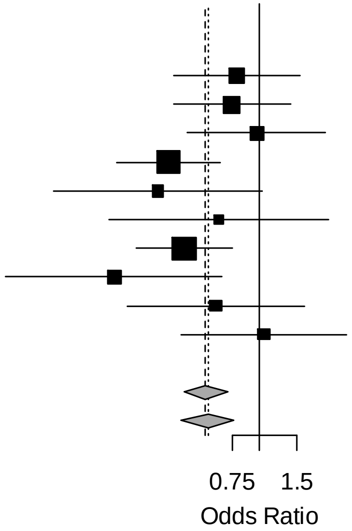

Placebo VS Venlafaxine : Remission

Study	Placebo Events	Placebo Total	Venlafaxine Events	Venlafaxine Total	OR	95%-CI	W(fixed)	W(random)
WXL101497	63	199	94	189	0.47	[0.31; 0.71]	23.2%	23.3%
AK130940	71	189	109	198	0.49	[0.33; 0.74]	23.4%	24.1%
Guelfi	6	47	12	46	0.41	[0.14; 1.22]	3.7%	3.4%
Thase	19	102	32	95	0.45	[0.23; 0.87]	9.5%	9.2%
Rudolph	18	98	35	100	0.42	[0.22; 0.81]	9.9%	9.2%
Nemeroff	22	102	31	102	0.63	[0.33; 1.19]	8.5%	9.9%
Sheehan	16	95	24	95	0.60	[0.29; 1.22]	7%	7.9%
Silverstone	28	118	56	122	0.37	[0.21; 0.64]	14.8%	13%
**Fixed effect model**	**243**	**950**	**393**	**947**	**0.47**	**[0.39; 0.58]**	**100%**	**--**
**Random effects model**					**0.47**	**[0.39; 0.58]**	**--**	**100%**

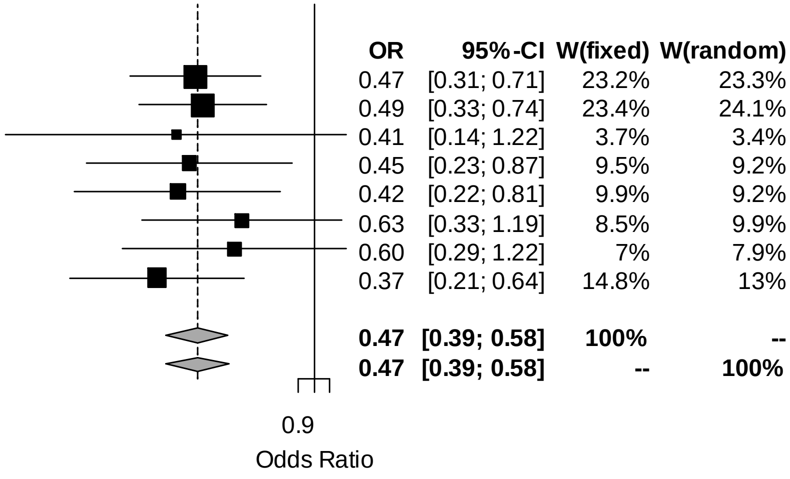

Fluoxetine VS Venlafaxine : Remission

Study	Fluoxetine Events	Fluoxetine Total	Venlafaxine Events	Venlafaxine Total	OR	95%-CI	W(fixed)	W(random)
Rudolph	23	103	35	100	0.53	[0.29; 0.99]	8.3%	7.8%
Nemeroff	28	104	31	102	0.84	[0.46; 1.54]	6.8%	8.1%
Sheehan	17	99	24	95	0.61	[0.31; 1.23]	6.1%	6.4%
Silverstone	54	119	56	122	0.98	[0.59; 1.63]	9%	10.8%
Alves	5	47	12	40	0.28	[0.09; 0.88]	3.5%	2.6%
Costa e Silva	112	186	118	196	1.00	[0.66; 1.51]	13.7%	14.9%
De Nayer	27	73	38	73	0.54	[0.28; 1.05]	7.2%	7%
Keller	132	267	380	781	1.03	[0.78; 1.36]	29.3%	23.9%
Tzanakaki	19	54	22	55	0.81	[0.37; 1.77]	4.2%	5.3%
Tylee	58	170	61	171	0.93	[0.60; 1.46]	12%	13.2%
**Fixed effect model**	**475**	**1222**	**777**	**1735**	**0.86**	**[0.74; 1.01]**	**100%**	**--**
**Random effects model**					**0.83**	**[0.69; 1.00]**	**--**	**100%**

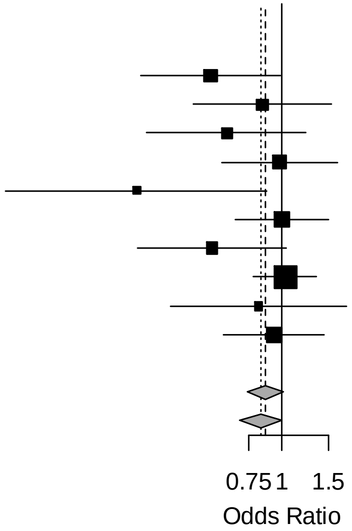

Supplement: Additional file 2 — FORESTPLOT. [file 1741-7015-12-106-S2.pdf]
